# Dosimetric analysis and biological evaluation between proton radiotherapy and photon radiotherapy for the long target of total esophageal squamous cell carcinoma

**DOI:** 10.3389/fonc.2022.954187

**Published:** 2022-10-03

**Authors:** Yongbin Cui, Yuteng Pan, Zhenjiang Li, Qiang Wu, Jingmin Zou, Dali Han, Yong Yin, Changsheng Ma

**Affiliations:** ^1^ Department of Radiation Oncology, Shandong Cancer Hospital and Institute, Shandong First Medical University and Shandong Academy of Medical Sciences, Jinan, China; ^2^ Medical Science and Technology Innovation Center, Shandong First Medical University and Shandong Academy of Medical Sciences, Jinan, China; ^3^ Affiliated Hospital of Weifang Medical University, School of Clinical Medicine, Weifang Medical University, Weifang, China

**Keywords:** proton radiotherapy, photon radiotherapy, dosimetric analysis, biological evaluation, esophageal squamous cell carcinoma

## Abstract

**Objective:**

The purpose of this study is to compare the dosimetric and biological evaluation differences between photon and proton radiation therapy.

**Methods:**

Thirty esophageal squamous cell carcinoma (ESCC) patients were generated for volumetric modulated arc therapy (VMAT) planning and intensity-modulated proton therapy (IMPT) planning to compare with intensity-modulated radiation therapy (IMRT) planning. According to dose–volume histogram (DVH), dose–volume parameters of the plan target volume (PTV) and homogeneity index (HI), conformity index (CI), and gradient index (GI) were used to analyze the differences between the various plans. For the organs at risk (OARS), dosimetric parameters were compared. Tumor control probability (TCP) and normal tissue complication probability (NTCP) was also used to evaluate the biological effectiveness of different plannings.

**Results:**

CI, HI, and GI of IMPT planning were significantly superior in the three types of planning (*p* < 0.001, *p* < 0.001, and *p* < 0.001, respectively). Compared to IMRT and VMAT planning, IMPT planning improved the TCP (*p*<0.001, *p*<0.001, respectively). As for OARs, IMPT reduced the bilateral lung and heart accepted irradiation dose and volume. The dosimetric parameters, such as mean lung dose (MLD), mean heart dose (MHD), *V*_5_, *V*_10_, and *V*_20_, were significantly lower than IMRT or VMAT. IMPT afforded a lower maximum dose (*D*_max_) of the spinal cord than the other two-photon plans. What’s more, the radiation pneumonia of the left lung, which was caused by IMPT, was lower than IMRT and VMAT. IMPT achieved the pericarditis probability of heart is only 1.73% ± 0.24%. For spinal cord myelitis necrosis, there was no significant difference between the three different technologies.

**Conclusion:**

Proton radiotherapy is an effective technology to relieve esophageal cancer, which could improve the TCP and spare the heart, lungs, and spinal cord. Our study provides a prediction of radiotherapy outcomes and further guides the individual treatment.

## Introduction

Esophageal cancer is one of the malignant tumors with the highest incidence worldwide (1). In East Asia, the subtype of esophageal cancer (EC) is mainly squamous cell carcinoma, with a poor prognosis and 5-year survival rates of less than 20% (2). As an effective treatment method for esophageal squamous cell carcinoma (ESCC), radiotherapy has been widely used in clinical therapy. Compared to two-dimensional conformal radiotherapy (2D-CRT), three-dimensional conformal radiotherapy (3D-CRT) can significantly improve the dose distribution in the target volume and reduce the accepted dose of normal tissues (3). However, compared to 3D-CRT, intensity-modulated radiotherapy (IMRT) has been used for the radiotherapy of ESCC patients because of its ability to provide superior target volume coverage, conformality, and ability to reduce dose to normal tissues (4). Recently, volume-modulated arc therapy (VMAT) for patients with ESCC has also been widely explored (5). Nonetheless, no matter whether IMRT or VMAT, photon radiotherapy will lead to normal tissue toxicity to some degree.

Hence, it is critical to ensure tumor control probability (TCP) while decreasing dose to normal tissues and normal tissue complication probability (NTCP). In proton beam, there is a deposition characteristic called “Bragg peak,” which can be used to create a matchable depth and thickness of the tumor target (6). Previous studies have demonstrated that proton therapy could provide a dose-sparing advantage for organs at risk (OARs) in lung cancer patients (7). Further studies have also proved that proton therapy has therapeutic advantages over conventional external radiotherapy in esophageal cancer (8).

However, whether it is photon therapy or proton therapy, the current research focuses on the dosimetric differences (9, 10). A few studies have looked into the differences in additional biological effects between photon and proton therapy. Wang et al. (11)developed and tested a Lyman–Kutcher–Burman (LKB) model to predict radiation esophagitis (RE) in nonsmall cell lung cancer (NSCLC) cancer. However, those NSCLC patients received passive-scattering proton therapy (PSBT) not modulated scanning.

Therefore, we aimed to compare the dosimetric difference between proton therapy and two-photon therapy in ESCC patients. Thereafter, TCP and NTCP prediction methods are used to predict the radiotherapy outcomes and toxicity. The purpose of this study is to compare the dosimetry advantages of intensity-modulated proton therapy (IMPT) compared with IMRT and VMAT in radiotherapy for patients with ESCC, and then predict the biological effects of TCP and NTCP to guide the individual radiotherapy.

## Materials and methods

### Patients and imaging acquisition

Thirty ESCC patients were recruited in Shandong Cancer Hospital and Institute, who received radiation therapy with the prescribed dose of 60 Gy between 2015 and 2020. Inclusion criteria were as follows: (1) patients with unresected esophageal cancer; (2) no prior history of radiotherapy and chemotherapy; and (3) no prior cardiac or respiratory diseases. Exclusion criteria were as follows: (1) changed treatment regimens during definitive radiotherapy. (2) a combination of other malignancies. This study was approved by the Ethics Committee of our institute according to the Declaration of Helsinki.

All patients were scanned with Philips Big Core CT (Phillips Medical Systems, 96 Highland Heights, OH). The scanning parameters were as follows: tube voltage: 120 KvP, tube current: 53–400 mA, each scanning period: 2.8 s, interval time: 1.8 s, scanning layer thickness: 5 mm, and a vacuum cushion was used to fix the scanning process. The scanned images were uploaded to the Eclipse15.5 treatment planning system (Varian Medical Systems, Palo Alto, CA, USA) for delineation of the target volume, OARs, and designing radiotherapy plans.

### Target volume and organ-at-risk delineation

The target volume and OARs were delineated by the same oncologist with more than 5 years of work experience. The gross target volume (GTV) was delineated on the target volume of primary esophageal cancer and possible positive lymph node based on diagnostic CT, esophagoscopy, and pathological reports. The clinical target volume (CTV) was based on GTV and the subclinical area of the tumor, taking into account factors such as respiratory movement and esophageal peristalsis. The plan target volume (PTV) was defined as the 6-mm margin of CTV, and the OARs were limited to 5 mm under the skin, including the left and right lungs, heart, and spinal cord.

### Designing treatment planning

Three types of plans were designed for each patient: IMRT, VMAT, and IMPT. All patients have been prescribed a dose of 60 Gy in 30 fractions, with a single fraction dose of 2 Gy. The dose limits for OARs were as follows (12): normal lung *V*_5_ (percentage of the normal lung volume irradiated with more than 5 Gy) <65%, *V*_20_ <25%; cardiac *V*_30_ <46%, and mean heart dose (MLD) of <26 Gy (the average dose did not exceed 26 Gy). The maximum point dose is less than 48 Gy in the spinal cord (*D*_max_ *<*48 Gy).

**Figure 1:**
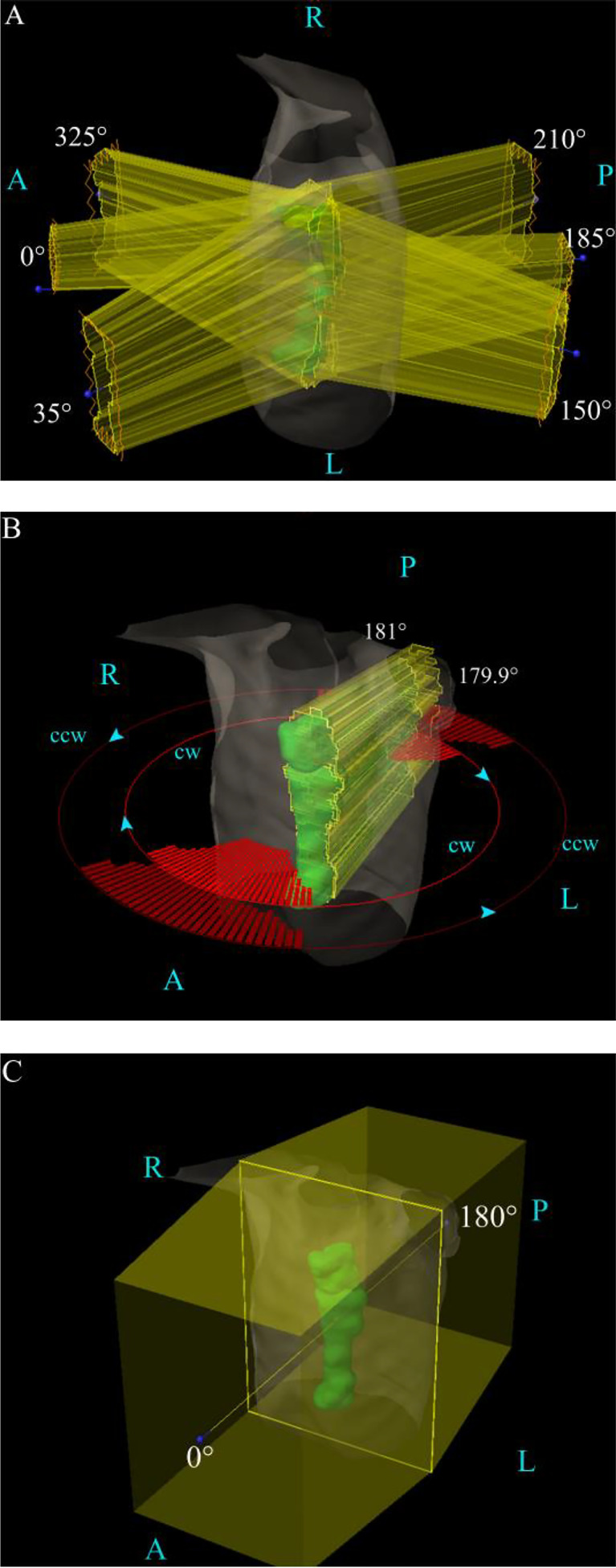
The field angle arrangement of the three various plans. **(A)** The IMRT plan’s field arrangement. **(B)** The VMAT plan’s field arrangement. **(C)** The IMPT plan’s field arrangement. A, anterior; L, left; P, posterior; R, right.

Among them, two-photon plans were designed based on Varian Eclipse15.5 TPS. The IMPT plan design was based on Varian Eclipse ProBeam proton systems. For the IMRT plan, as shown in **Figure 1A**, the use of 6 fields was 0°, 35°,150°,185°, 210°, and 325°, respectively. For the VMAT plan, we used 179.9° CCW 181° and 181° CW 179.9°, in order to protect the lungs, setting the angle to avoid 150°–30°, 330°–210°, 210°–330°, and 30°–150°, as shown in **Figure 1B**.

For the IMPT plan, the Varian Eclipse ProBeam Proton system utilizes the anteroposterior technique (0°/180°), as shown in **Figure 1C**. The nonlinear general proton optimizer (NUPO) algorithm was used to generate the plan, and the proton convolution superposition algorithm with a grid size of 0.25 cm was used to calculate the dose planning optimization, taking into account the positioning error of 3 mm and the range uncertainty of 3.5%. The beam output was determined using a relative biologic effectiveness (RBE) of 1.1 and is specified in cobalt gray equivalent (CGE) units (13).

### Dosimetric analysis

Dose-volume parameters of PTV were obtained from DVH: dose received by 2% of the target volume (*D*_2_), dose received by 98% of the target volume (*D*_98_), maximum dose (*D*_max_), mean dose (*D*_mean_), minimum dose (*D*_min_), CI, HI, and GI. The parameters used to evaluate the OAR sparing include the following: MLD, *V*_5_, *V*_10_, *V*_15_, and *V*_20_ of the bilateral lung; MHD, *V*_5_, *V*_10_, *V*_15_, *V*_20_, *V*_30_, and *V*_40_ of the heart (*V_X_* represents the volume percentage receiving more than *X* Gy); and the *D*_max_ and *D*_mean_ of the spinal cord.

The CI was calculated according to the following formula:


(1) $$\text{CI }=\;\left(\frac{V_{\text{TR}}}{V_{T}}\right)\times \left(\frac{V_{\text{TR}}}{V_{T}}\right)$$

The CI ranged from 0 to 1, where 1 indicated perfect overlap (identical structures). A value near 0 indicated the total absence of conformation; the target volume was not irradiated.

*V*_TR_ is the volume of the reference isodose curve coverage of the PTV, *V_T_* is the volume of the PTV, *V_R_* is the volume of the reference isodose curve coverage of the body (including PTV), and 95% of the prescription dose is defined as the reference isodose curve.

The homogeneity index was calculated according to the following formula:


(2) $$\text{HI }=\;\frac{D_{2\;}-\;D_{98}}{D_{50\;}}$$

The HI ranged from 0 to 1, where 0 was the ideal value. A higher HI indicates poorer homogeneity.

*D*_2_ is the dose received by 2% of the target volume, *D*_98_ is the dose received by 98% of the target volume, and *D*_50_ is the dose received by 50% of the target volume.

The gradient index was calculated according to the following formula:


(3) $$\text{GI }=\frac{V_{50}}{V_{100}}$$

In particular, *V*_50_ represents the volume receiving at least 50% of the prescription dose. *V*_100_ represents the volume receiving at least 100% of the prescription dose.

### TCP and NTCP evaluation

The TCP of PTV and NTCP of the left and right lungs, heart, and spinal cord were used to evaluate radiotherapy plans and predict organ’s toxicity. The TCP and NTCP were calculated based on MATLAB R2013b (www.mathworks.com, The MathWorks Inc., Natick, MA, USA).

The TCP calculation formula was based on the equivalent uniform dose (EUD) model (14). The TCP formula and EUD model are as follows:


(4) $$\text{TCP }=\;\frac{1}{1＋（\frac{\text{TC}D_{50}}{\text{EUD}}{)}^{4\gamma_{50}}}$$

TCD_50_ is the dose required when the TCP is 50%, and γ_50_ is the slope of the dose–response curve when the tumor target control rate is 50%.


(5) $$\text{EUD }=\;{\left(\;\sum_{i=1}({\text{ViDi}}^{a})\right)}^{\frac{1}{a}}$$

*a* is a unit-free parameter describing the volume effect size of the tumor or normal structure; *V_i_* is the relative volume related to dose-voxel *D_i_*. In patients with esophageal squamous cell carcinoma treated with radiotherapy, TCD_50_ is 51.24 Gy, γ_50_ is 0.83, and *a* is 0.3.

The calculation of NTCP for the probability of normal tissue complications of OARs is based on the Lyman–Kutcher–Burman (LKB) model (14–17), and the calculation formula is as follows:


(6) $$\text{NTCP }=\;\frac{1}{1＋{\left(\frac{\text{TD}50}{\text{EUD}}\right)}^{4\gamma_{50}\;}}$$

TD_50_ represents the dose to the whole organ (or reference volume), which will result in a 50% probability of complications. γ_50_ is a unitless model parameter that is specific to the normal structure or tumor of interest and describes the slope of the dose–response curve. Parameters *a* and γ_50_ should be obtained by fitting clinical dose–response data with EUD-based NTCP or EUD-based TCP model (14, 18).

In the calculation of TCP and NTCP, the EQD_2_ (19, 20) formula is used for fractional correction based on voxels. EQD_2_ is the bioequivalent physical dose, and the unit is 2 Gy/min of partial volume *V_i_*. The formula is as follows:


(7) $$\text{EQD}2\;=\;D_{i}\left(1+\frac{{\text{d}}_{i}}{\text{α}/\text{β}}\right)$$

*D_i_* is the total absorbed dose in the reference treatment plan, *d_i_* is the dose of each subdose in the treatment process, and α/β is the tissue-specific LQ parameter of exposed organs (19, 21).

The predicted clinical endpoint of the lung is radiation pneumonitis, and pericarditis of any grade is the endpoint of the heart. For the spinal cord, spinal cord myelitis necrosis is the predicted endpoint. For TCP prediction, the parameters published by Niemierko (20) were adopted. For pneumonitis, the parameters published by Seppenwoolde (22) were adopted. For pericarditis of any grade, the parameters published by Gagliardi (23) were adopted. For spinal cord myelitis necrosis, the parameters published by Agren (24, 25) were adopted. All the parameters are shown in **Table 1**.

**Table 1:** The parameters of the formulas.

	TCP	NTCP lung	NTCP heart	NTCP spinal cord
TCD_50_ (Gy)	51.24			
TD_50_ (Gy)		34	50.6	68.6
α/β	10	2	2.5	2
a	0.3	3	2.5	13
γ_50_	0.83	0.9	1.3	1.9

TCP, tumor control probability; NTCP lung, normal tissue complication probability of the lungs; NTCP heart, normal tissue complication probability of the heart; NTCP spinal cord, normal tissue complication probability of spinal cord.

### Statistical analysis

TCP and NTCP were calculated based on MATLAB2013b (The MathWorks, Natick, MA, USA), and SPSS 25.0 was used for data analysis (IBM Corp, Armonk, NY, USA). All the results were presented in the form of mean ± standard deviation. Univariate ANOVA analysis and Tukey were used to conduct a *post-hoc t*-test between the three plans. *p*-values of less than 0.05 were considered statistically different.

## Results

Thirty patients with ESCC who underwent radiotherapy achieved the expected clinical dose limits for all types of plans. The detailed values of PTV’s dose-volume parameters are shown in **Table 2**. The parameters include the following: *D*_2_, *D*_98_, *D*_max_, *D*_mean_, *D*_min_, CI, HI, and GI all met clinical requirements, but there were significant differences among the three planning methods in *D*_2_, *D*_98_, *D*_mean_, *D*_min_, CI, HI, and GI. The CI of IMPT was 0.89 ± 0.04, which was higher than that of IMRT (0.85 ± 0.03) and VMAT (0.65 ± 0.20). The GI of the IMPT plan was 2.23 ± 0.30, which was significantly lower than IMRT (5.50 ± 1.27) and VMAT (3.60 ± 0.60).

**Table 2:** The dose-volume parameters of PTV.

Parameters	IMRT	VMAT	IMPT	ANOVA *p-*value	*p-*values		
					IMRT versus VMAT	IMRT versus IMPT	VMAT versus IMPT
*D*_2_ (Gy)	65.25 ± 0.70	66.69 ± 1.23	65.21 ± 0.51	0.001	0.003	0.996	0.002
*D*_98_ (Gy)	59.93 ± 0.76	57.87 ± 0.88	60.28 ± 0.82	0.000	0.000	0.606	0.000
*D*_max_ (Gy)	67.57 ± 0.93	69.54 ± 2.24	69.11 ± 2.12	0.061	0.063	0.170	0.866
*D*_mean_ (Gy)	63.09 ± 0.21	63.66 ± 0.68	63.16 ± 0.01	0.009	0.012	0.928	0.030
*D*_min_ (Gy)	39.9 ± 10.67	35.80 ± 6.36	47.09 ± 7.23	0.017	0.508	0.153	0.014
CI	0.85 ± 0.03	0.65 ± 0.20	0.89 ± 0.04	0.000	0.003	0.746	0.000
HI	0.08 ± 0.02	0.14 ± 0.03	0.08 ± 0.02	0.000	0.000	0.817	0.000
GI	5.50 ± 1.27	3.60 ± 0.60	2.23 ± 0.30	0.000	0.000	0.000	0.003

All values are shown as mean ± standard deviation.

IMRT, intensity-modulated radiation therapy; VMAT, volumetric-modulated arc therapy; IMPT, intensity-modulated radiation therapy; D_2_, dose received by 2% of the target volume; D_98_, dose received by 98% of the target volume; D_mean_, the mean dose of PTV; D_max_, the maximum dose; D_min_, the minimum dose; CI, conformity index; HI, homogeneity index; GI, gradient index.

The dose-volume parameters of OARs are summarized in **Table 3**. As shown in **Table 3**, the IMPT plan showed significant protection of OARs, such as the lungs and heart. For the left and right lungs, the MLD, *V*_5_, *V*_10_, and *V*_15_ of IMPT were significantly lower than the IMRT and VMAT; there was no significant difference between IMRT and VMAT (*p* > 0.05). For the heart, MHD, *V*_10_, and *V*_20_ of IMPT were significantly lower than IMRT (*p* = 0.048, *p* = 0.049, *p* = 0.008, respectively). MHD, *V*_20_, *V*_30_, and *V*_40_ of the IMPT plan were also significantly lower than the VMAT plan (*p* = 0.011, *p* = 0.006, *p* = 0.008, *p* = 0.016, respectively), while there was no significant difference between the IMRT and VMAT plan. For the spinal cord, the *D*_max_ of the IMPT plan was significantly lower than VMAT (*p* = 0.001), while there was no difference between IMPT and IMRT. Furthermore, the *D*_mean_ of the spinal cord also showed no significant difference among the three plans.

**Table 3:** The dose-volume parameters of OARs.

OARs	IMRT	VMAT	IMPT	ANOVA *p-*value	*p*-value		
					IMRT versus VMAT	IMRT versus IMPT	VMAT versus IMPT
Right lung							
MLD (Gy)	11.78 ± 3.78	10.79 ± 3.75	4.08 ± 1.94	0.000	0.780	0.000	0.000
*V*_5_ (%)	52.85 ± 19.21	55.29 ± 19.66	13.02 ± 5.64	0.000	0.940	0.000	0.000
*V*_10_ (%)	35.59 ± 15.36	32.45 ± 18.73	10.79 ± 4.91	0.001	0.876	0.002	0.006
*V*_15_ (%)	27.08 ± 3.48	21.93 ± 4.01	9.38 ± 1.40	0.002	0.495	0.001	0.025
*V*_20_ (%)	21.40 ± 7.93	15.77 ± 8.45	8.27 ± 4.04	0.001	0.196	0.001	0.063
Left lung							
MLD (Gy)	13.35 ± 3.68	12.20 ± 3.08	4.31 ± 1.85	0.000	0.668	0.000	0.000
*V*_5_ (%)	57.17 ± 17.63	60.72 ± 15.77	15.41 ± 4.94	0.000	0.837	0.000	0.000
*V*_10_ (%)	40.98 ± 14.19	38.84 ± 14.92	12.53 ± 4.62	0.000	0.919	0.000	0.000
*V*_15_ (%)	32.70 ± 10.63	26.86 ± 9.40	10.67 ± 4.42	0.000	0.296	0.000	0.000
*V*_20_ (%)	26.91 ± 8.09	19.35 ± 7.15	9.16 ± 4.22	0.000	0.045	0.000	0.006
Heart							
MHD (Gy)	21.90 ± 9.58	24.90 ± 14.28	10.35 ± 4.86	0.010	0.793	0.048	0.011
*V*_5_ (%)	75.70 ± 32.09	73.45 ± 33.55	46.22 ± 23.46	0.067	0.985	0.090	0.125
*V*_10_ (%)	67.23 ± 30.84	64.19 ± 31.73	36.31 ± 18.89	0.036	0.968	0.049	0.082
*V*_20_ (%)	49.71 ± 25.31	50.89 ± 28.31	16.83 ± 8.68	0.003	0.992	0.008	0.006
*V*_30_ (%)	30.82 ± 16.73	38.31 ± 28.84	9.97 ± 5.57	0.009	0.671	0.061	0.008
*V*_40_ (%)	16.80 ± 9.62	28.23 ± 25.63	6.90 ± 4.18	0.021	0.263	0.363	0.016
Spinal cord							
*D*_max_ (Gy)	44.96 ± 3.31	49.12 ± 6.83	40.2 ± 3.01	0.001	0.139	0.079	0.001
*D*_mean_ (Gy)	20.95 ± 8.33	23.25 ± 10.09	15.72 ± 6.20	0.138	0.813	0.356	0.128

All data are displayed as mean ± standard deviation.

OARs, organ at risks; MLD, mean lung dose; MHD, mean heart dose; Vx, V_X_ represents the volume percentage receiving more than X Gy OARs.

**Table 4** shows the TCP of PTV and the NTCP of OARs. The TCP of the IMPT plan was 73.92% ± 0.01%, which was significantly higher than IMRT (67.28% ± 0.35%) and VMAT (67.92% ± 0.89%) (*p* < 0.001 and *p* < 0.001, respectively). The NTCP for right lung radiation pneumonia and left lung radiation pneumonia of IMPT were 12.99% ± 8.43% and 10.23% ± 7.44%, respectively. Although there was no statistical difference, especially in the left lung, the NTCP of the IMPT plan was 3.78% and 3.89% lower than IMRT and VMAT, respectively. As for the NTCP of the spinal cord, VMAT was significantly higher than IMPT (*p* = 0.016).

**Table 4:** TCP and NTCP.

	IMRT	VMAT	IMPT	ANOVA *p-*values	*p*-values		
					IMRT *vs*. VMAT	IMRT *vs*. IMPT	VMAT *vs* IMPT
TCP_PTV_ (%)	67.28 ± 0.35	67.97 ± 0.89	73.92 ± 0.01	0.000	0.028	0.000	0.000
NTCP_Right lung_ (%)	13.28 ± 6.29	12.82 ± 7.47	12.99 ± 8.43	0.990	0.990	0.996	0.999
NTCP_Left lung_ (%)	14.01 ± 6.67	14.12 ± 8.94	10.23 ± 7.44	0.451	0.999	0.526	0.507
NTCP_heart_ (%)	4.64 ± 5.07	21.22 ± 24.80	1.73 ± 2.24	0.013	0.045	0.897	0.016
NTCP_spinal cord_ (%)	0.07 ± 0.13	0.27 ± 0.29	0.12 ± 0.21	0.015	0.066	0.810	0.016

All data are displayed as mean ± standard deviation.

TCP, tumor control probability; NTCP, normal tissue complication probability.

## Discussion

Although photon radiotherapy has been widely used in the clinical treatment of ESCC patients, its late toxicity to normal tissues is still an urgent problem to be solved (26), such as radiation pneumonia, pericarditis, myelitis, etc. In our study, by dosimetric analysis and biological effect evaluation, we conclude that IMPT has the advantage of treating with ESCC. Compared with conventional photon radiotherapy techniques such as IMRT and VMAT, IMPT could significantly reduce the dose and volume of radiation to the heart, lungs, and spinal cord. While improving the TCP, it could provide superior protection for the heart and lungs (especially the left lung).

Some studies have shown that the proton beam has a high response to tumor cells and proton therapy could improve the TCP (6, 27), which is consistent with our research results. Comparing the three treatment technologies, our predicted results show that proton therapy has the highest TCP among the three groups. This might be related to the high linear energy transfer (LET) of the proton beam. LET is a commonly used method to indicate the radiation mass of the ion beam. Generally, high LET is associated with an increase in relative biological effectiveness (RBE) (13, 28). Also, RBE is assumed as 1.1 (13) in our study.

In fact, some studies have investigated the dosimetric and radiobiological differences between photon and proton therapy (29–32). Stokkevag et al. (29) evaluated the differences between proton planning and VMAT planning for children with brain tumors. Based on the LKB model, the NTCP values were compared with the two different plannings. As for the model’s parameters, they found that there was no difference between adult and pediatric populations. The parameters were also used in the LKB model for the two different planning comparisons. They found proton therapy planning significantly reduced the auditory complications, xerostomia, and risk of secondary cancers of the brain and salivary glands. As for liver cancer, Prayongrat et al. (30) used the NTCP model to predict the probability of radiation-induced liver toxicity (RILT). They also confirmed the estimated NTCP and ΔNTCP for individual patients along with consideration of uncertainties improving the reliability of the NTCP model-based approach. Feng et al. (31) compared the biological effects of two different beam angle configurations of IMPT. From the prediction of the NTCP model, they concluded that the IMPT planning with superior–inferior oblique posterior beams had a better spare of liver, heart, and lungs at the slight cost of spinal cord maximum dose protection. Recently, Liu et al. (32) investigated the dosimetric and potential clinical benefits for locally advanced pancreatic cancer treated with proton beam therapy. As for the clinical benefits, they also applied the NTCP models and derived the parameters from the previous photon studies. The results demonstrated that two-field IMPT provided lower severe toxicity for the stomach and duodenum than VMAT. Although there were no special models and parameters designed for proton therapy, those studies have proved that the model could provide a reference for radiotherapy (including proton therapy) and further guided radiotherapy planning designing and choosing.

As for the ESCC, there are many important organs around the esophagus, such as the heart, lungs, spinal cone (even ribs), and thymus. Currently, it has been proved that proton therapy can significantly spare the dose of lung and heart, such as MLD, *V*_5_, *V*_10_, and *V*_20_ of the lung (33). This result is consistent with our study. We also found that due to the left physiological laterality of the esophagus, photon therapy would cause the left lung to receive a higher dose. While the protection of the left lung is advantageous in the proton therapy plan. The NTCP of radiation pneumonia in the left lung is 3.98% and 3.98% lower than IMRT and VMAT, respectively. The incidence of acute cardiac events is thought to be related to the dose received by the heart (26, 34). Keiichi et al. (35) stated in the article that radiation caused cardiotoxicity and increased the incidence of acute cardiac events. Wang et al. (36) also demonstrated that the incidence of cardiac complications after proton radiation therapy was significantly lower than that of photon radiation therapy. This conclusion is in consonance with our study that showed proton therapy can significantly spare the radiation dose and volume of the heart, and the NTCP of cardiac pericarditis is the lowest among the three treatments, achieving the protection of the heart during radiotherapy.

There are several limitations to our study. First, the sample size of the study is not large enough, and the follow-up needs to be demonstrated by a large cohort study. Second, when discussing OARs, the heart is only taken as a whole structure without specific analysis of substructures. However, Shiraishi et al. (37) have discussed and concluded that the radiation exposure of PBT to the whole heart and cardiac substructures was significantly lower than the IMRT plan. Finally, this study does not find a suitable prediction model for other OARs, which may be needed for further study.

## Conclusion

This study demonstrated that the IMPT could effectively spare the heart and lungs and reduce the irradiation dose and coverage volume. Furthermore, IMPT is able to improve the TCP of ESCC significantly, which might change the outcome directly. To some degree, the IMPT plan will decrease the NTCP of the heart and left lung. The prediction of TCP and NTCP could also provide a reference and guide future individual treatment.

## Data availability statement

The raw data supporting the conclusions of this article will be made available by the authors, without undue reservation.

## Ethics statement

This study was reviewed and approved by Shandong Cancer Hospital and Institute Ethics Committee. Written informed consent for participation was not required for this study in accordance with the national legislation and the institutional requirements.

## Author contributions

YC drafted the concept and the manuscript. YP, ZL, QW and JZ contributed to acquiring, analyzing, and interpreting data. DH contributed to the target volume delineation and radiotherapy planning examination. YY and CM contributed to acquiring data, designing the study, and enhancing its intellectual content. All authors contributed to the article and approved the submitted version.

## Funding

This work is supported by the National Nature Science Foundation of China (81800156, 81974467), Shandong Province Key R&D Program (2018GSF118031), the Natural Science Foundation of Shandong Province (ZR2019MH136), and the Taishan Scholars Project of Shandong Province (ts201712098).

## Acknowledgments

The authors would like to thank the editor and reviewers for their insightful suggestions, which helped improve the manuscript.

## Conflict of interest

The authors declare that the research was conducted in the absence of any commercial or financial relationships that could be construed as a potential conflict of interest.

## Publisher’s note

All claims expressed in this article are solely those of the authors and do not necessarily represent those of their affiliated organizations, or those of the publisher, the editors and the reviewers. Any product that may be evaluated in this article, or claim that may be made by its manufacturer, is not guaranteed or endorsed by the publisher.
